# Carbogenic nanodots derived from organo-templated zeolites with modulated full-color luminescence†
†Electronic supplementary information (ESI) available: PXRD, ^13^C MAS NMR spectra, TEM of CNDs@MgAPO-44, schematic diagram of **CHA** cage, ^27^Al MAS NMR spectra, FTIR spectra, PL emission spectra, cellular imaging, excitation–emission matrix for Ref-CNDs, UV-vis absorption spectra, PL decay curves and their fitting parameters. See DOI: 10.1039/c6sc00085a


**DOI:** 10.1039/c6sc00085a

**Published:** 2016-02-12

**Authors:** Ying Mu, Ning Wang, Zaicheng Sun, Jing Wang, Jiyang Li, Jihong Yu

**Affiliations:** a State Key Laboratory of Inorganic Synthesis and Preparative Chemistry , College of Chemistry , Jilin University , Changchun 130012 , P. R. China . Email: lijiyang@jlu.edu.cn ; Email: jihong@jlu.edu.cn ; Fax: +86-431-8516-8608 ; Tel: +86-431-8516-8608; b Beijing Key Laboratory for Green Catalysis and Separation , Department of Chemistry and Chemical Engineering , Beijing University of Technology , Beijing , 100124 , P. R. China

## Abstract

Hydrophilic N-doped carbogenic nanodots (denoted as CNDs) have been prepared from a *N*-methylpiperazine-templated zeolite precursor by calcination and NaOH treatment. The isolated CNDs exhibit tunable photoluminescence according to the concentration and pH value of aqueous dispersions of the CNDs. Fine-tuning of the fluorescence emission wavelength across the entire visible spectrum can be easily achieved by varying the concentration of the CND dispersions. Meanwhile, both the emission wavelength and intensity of the photoluminescence can be tuned by controlling the pH value of the CND dispersion. The pyrolysis of organic templates confined in nanoporous zeolites represents a new approach to controlling the optical properties of CNDs, which may open more opportunities in applications such as multimodal sensing and full-color displays.

## Abstract 2

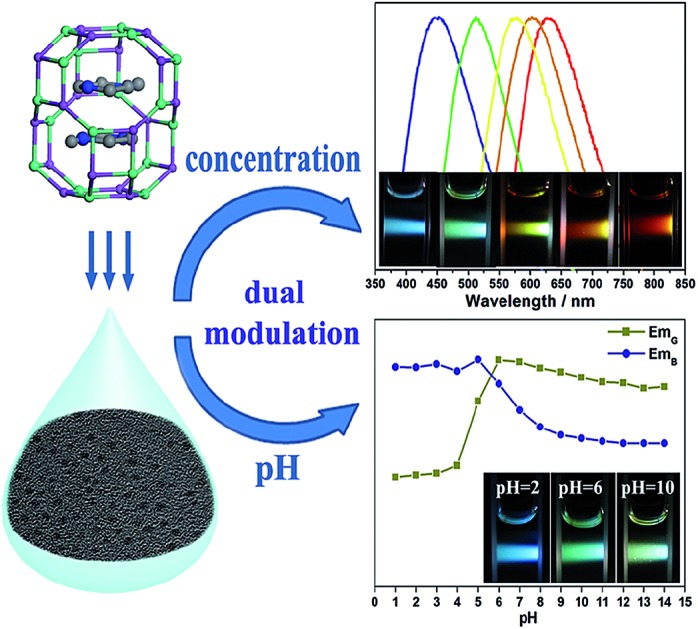
Dual modulated luminescence of carbogenic nanodots derived from zeolites has been acquired by controlling the concentration and pH value of CND aqueous dispersions.

## Introduction

Carbogenic nanodots (denoted as CNDs) are defined as small graphene-based oxygenous carbon nanoparticles with a size of less than 10 nm. As a new member of the nanocarbon family, luminescent CNDs have attracted increasing attention due to their intriguing advantages when compared with conventional semiconductor quantum dots, such as their low toxicity, biocompatibility, low cost and chemical inertness.^1^–^3^ Thus, CNDs have broad potential applications in bioimaging, chemical sensing, drug delivery, photocatalysis and multicolor patterning.^4^–^9^ The optical properties of CNDs are closely associated with their preparation methods. To date, various methods such as arc discharge,^1^ laser ablation,^10^ electrochemical oxidation,^11^–^13^ microwave,^14^,^15^ hydrothermal method,^5^,^16^,^17^ ultrasonic,^18^ combustion,^19^,^20^ *etc.*, have been utilized to prepare CNDs from suitable carbon sources or molecular precursors. Recently, the pyrolysis of organic templates confined within zeolites has been found to be an efficient approach for preparing well dispersed CNDs with uniform size, benefiting from the well-confined pore space of zeolites.^21^–^23^ So far, over 200 types of zeolite structures have been discovered and hundreds of organic templates containing N, P and S species have been used in zeolite synthesis.^24^–^26^ The diverse nanoporous architectures of the host zeolites and different organic templates may provide increased opportunities to prepare CNDs with variable composition, size and distinct surface chemistry, and CNDs with interesting luminescence properties may be prepared using this method. Nevertheless, research in this regard is still limited.

Tunable photoluminescence (PL) is one of the attractive properties of CNDs for their practical application, in particular, CNDs with full-color emissions are highly desirable.^4^,^27^,^28^ Although most of the reported CNDs possess excitation-dependent fluorescence behaviour, their fluorescence emissions usually fall within a relatively narrow wavelength (100–150 nm), accompanied by significant decreases in their fluorescence intensities.^29^–^32^ Considering many factors, such as particle size, chemical composition, surface groups, pH value, *etc.*, may affect the PL properties of CNDs, some alternative methods have been used to adjust the PL of CNDs. For example, Li *et al.* have reported the size-dependent photoluminescence of CNDs by controlling the current intensity of an electrochemical oxidation method.^11^ Zhu *et al.* have reported the PL modulation of graphene quantum dots through a surface chemistry route.^33^ Liu *et al.* have observed that the fluorescence intensity of CNDs decreased when the pH value of the solution was shifted from 7.0 - regardless of whether it was increased or decreased.^19^ In contrast, Krysmann *et al.* observed that the emission intensities of CNDs were similar within the pH range of 4–12, but decreased abruptly at lower pH values (<4).^32^ Recently, Nie *et al.* and Hu *et al.* have presented CNDs with full-color emissions by varying the reagents and reaction conditions.^34^,^35^ The exploitation of simple and effective means to achieve the modulation of PL of CNDs with full color emissions is considerably interesting.

In this work, we present the preparation of uniform N-doped CNDs by the pyrolysis of *N*-methylpiperazine (NMP) confined in zeolite MgAPO-44 with **CHA** zeotype topology. The isolated CNDs exhibit excellent aqueous dispersibility and stability. Interestingly, the PL emission of aqueous dispersions of the CNDs can be simply modulated by controlling the concentration of CNDs, which display full-color emissions. In addition, not only the intensity of the photoluminescence, but also the emission wavelength can be tuned by varying the pH value of the CND dispersions. This ability to control the optical properties of CNDs may offer more opportunities in applications such as multimodal sensing and full-color displays.

## Experimental section

### Synthesis of the MgAPO-44 zeolite

The reagents and solvents employed in the synthesis were commercially available and used as received without further purification. The magnesium aluminophosphate zeolite MgAPO-44 was prepared under hydrothermal conditions in a reaction system of MgO–Al_2_O_3_–P_2_O_5_–NMP–H_2_O. Typically, pseudoboehmite (Al_2_O_3_, 62.5%) and magnesium acetate tetrahydrate (Mg(CH_3_COO)_2_·4H_2_O, 99.0%) were dispersed in a solution of orthophosphoric acid (H_3_PO_4_, 85 wt%) in water under vigorous stirring at room temperature. Then, NMP (C_5_H_12_N_2_, 99.0%) was added to this mixture. After stirring for one hour, a homogeneous gel with an overall molar composition of 1.0 Al_2_O_3_ : 1.0 MgO : 2.2 P_2_O_5_ : 5.0 NMP : 350 H_2_O was formed, which was heated under autogenous pressure at 180 °C in a 15 mL Teflon-lined stainless steel autoclave for 3 days. The crystals were washed in distilled water and dried at 60 °C overnight.

### Preparation and separation of CNDs

The as-synthesized zeolite product was placed into a normal furnace and heated from room temperature to 400 °C at a temperature ramp rate of 2 °C min^–1^ in air, followed by a 4 h isothermal hold at this temperature. Carbonization of NMP was occurring during this time, resulting in a CNDs@MgAPO-44 composite material.

400 mg of CNDs@MgAPO-44 sample was dissolved in 2.5 mL of 2.5 M NaOH aqueous solution at room temperature, and ultrasonic treatment was used to accelerate the dissolution. Then 5 mL of deionized water was added and insoluble residues were centrifugally separated from the solution. The dark colored supernatant was collected and neutralised with HCl solution to a pH value of about 7, it was then further centrifugally separated and purified by dialyzing with a cellulose ester membrane bag (molecular-weight cutoff = 1000). The initial purified CNDs were denoted as R-CNDs with a concentration of around 1.36 g L^–1^. The R-CNDs sample was diluted to 0.92, 0.68, 0.22 and 0.04 g L^–1^ samples (denoted as O-, Y-, G- and B-CNDs respectively). To study the influence of the pH value on the PL properties of the CNDs, NaOH and HCl aqueous solutions were used to adjust the pH value of the G-CND solution to 8–14 and 1–6, respectively.

### Characterization

Powder X-ray diffraction (PXRD) data were collected on a Rigaku Ultima IV diffractometer. The CND ethanol solution was spotted onto a salt plate and the spectrum was measured on a Bruker FTIR IFS-66V/S. A baseline correction was applied after measurement. UV-vis adsorption spectra were obtained on a Shimadzu UV-2550 spectrophotometer. The TEM and HRTEM images were taken on a FEI Tecnai G2 S-Twin F20 transmission electron microscope. The X-ray photoelectron spectroscopy (XPS) measurements were performed using a Thermo Escalab 250 spectrometer with monochromatized Al Kα excitation. ^13^C solid-state MAS NMR were performed on an Infinity Plus400 spectrometer operating at *B*_0_ = 9.7 T. ^27^Al solid-state MAS NMR were recorded on a Bruker AVANCE III 400 WB spectrometer operating at *B*_0_ = 9.4 T. Total Organic Carbon (TOC) analysis was performed on an Elementar Vario TOC cube. The absolute fluorescence quantum yields were measured on an Edinburgh FLS920 fluorimeter. Photoluminescence spectra were recorded on a Fluoromax-4 spectrofluorometer (Horiba Jobin-Yvon). Nano-second fluorescence lifetime measurements were performed using a time-correlated single-photon counting (TCSPC) system under right-angle sample geometry. A NanoLED-340 (Horiba, repetition rate 1 MHz) was used to excite the samples. The white light used in this work was obtained by irradiation with a High Power Xenon Lightsource (HPX-2000).

## Results and discussion

### Structures of the MgAPO-44 zeolite and CNDs@MgAPO-44

The powder X-ray diffraction (PXRD) pattern of MgAPO-44 (Fig. S1, ESI†) shows that the as-synthesized product is pure phase and has a **CHA** zeotype structure.^36^ When MgAPO-44 was calcined at 400 °C for 4 h, the MgAPO-44 framework was kept intact but its crystallinity decreased. ^13^C MAS NMR (Fig. S2, ESI†) analysis indicates the presence of a graphite-like carbon structure in the calcined sample. A transmission electronic microscopy (TEM) image of the calcined sample provides evidence for the existence of CNDs with a uniform size of about 2.5 nm (Fig. S3, ESI†). Since the cavity size of the **CHA** framework (about 0.94 nm in diameter, Fig. S4, ESI†) is much smaller than that of the CNDs, the CNDs might have been located in larger cavities formed due to the breakage of some bonds upon calcination. The ^27^Al MAS NMR study shows that four-coordinated Al atoms predominantly exist in MgAPO-44, but after calcination some octahedrally-coordinated Al atoms appear in CNDs@MgAPO-44 (Fig. S5, ESI†).

### Characterization of CNDs in aqueous dispersions

**Fig. 1:**
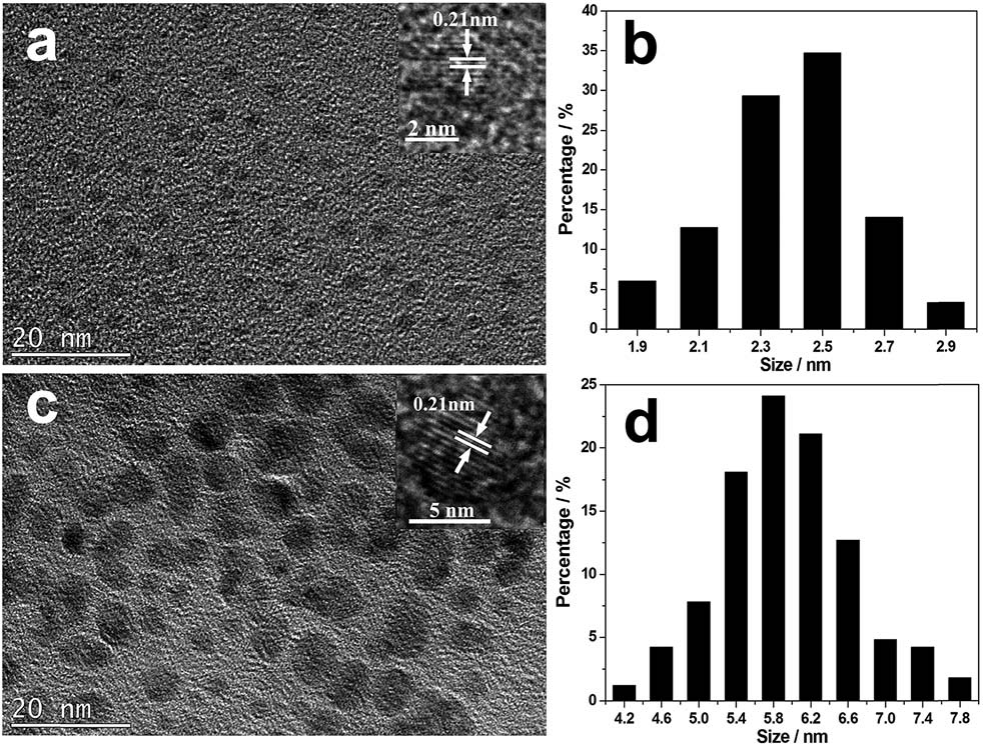
TEM and HRTEM images of the (a) B-CNDs and (c) R-CNDs and size histograms of the (b) B-CNDs and (d) R-CNDs obtained by counting about 150 particles.

TEM images of the isolated CNDs are shown in Fig. 1. They show that B-CNDs with a concentration of 0.04 g L^–1^ are monodispersed and have a uniform size of approximately 2.5 nm, which is consistent with the observed particle size of CNDs confined in a zeolite host. In contrast, the R-CNDs with a concentration of 1.36 g L^–1^ are partly agglomerated and the size distribution has a wider range with an average value of 5.8 nm. The high resolution TEM (HRTEM) images reveal their lattice spacings to be 0.21 nm, which is consistent with the lattice spacing of the (100) plane of graphene.^2^,^37^

**Fig. 2:**
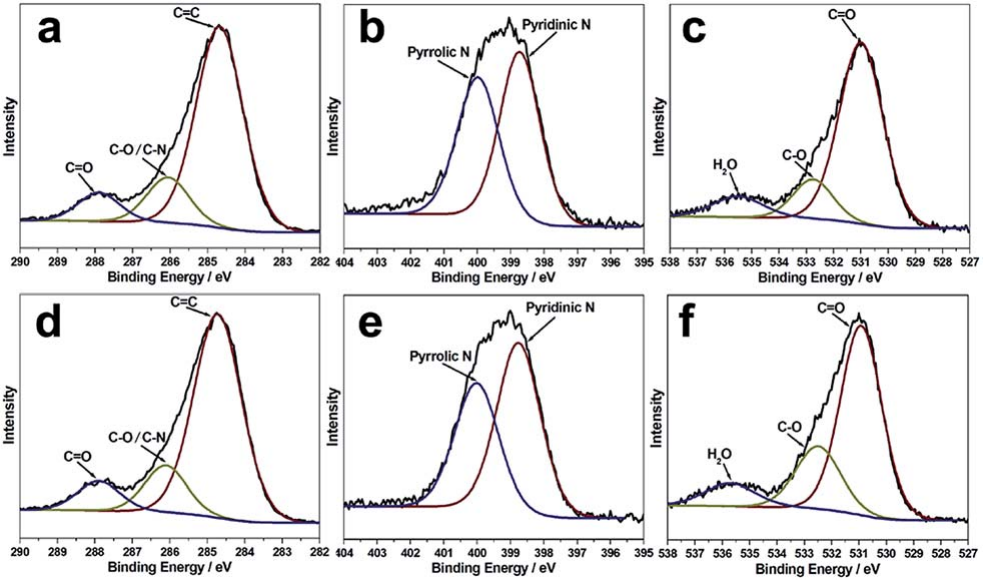
The C 1s, N 1s and O 1s XPS spectra for the R-CNDs (a–c) and B-CNDs (d–f).

The X-ray photoelectron (XPS) spectra of the B-CNDs and R-CNDs are shown in Fig. 2. The C 1s spectra can be deconvoluted into three peaks, which are attributed to the graphitic C=C bonds (284.7 eV), C–N/C–O bonds (286.2 eV) and C=O bonds (287.9 eV). The N 1s spectra can be deconvoluted into two peaks of pyridinic type (399.0 eV) and pyrrolic type (400.1 eV) N atoms. The O 1s spectra are assigned to O=C bonds (531.0 eV), O–C bonds (532.8 eV) and adsorbed water (535.6 eV).^4^,^37^ These results indicate that the structures and compositions of the R-CNDs and B-CNDs are similar, and both possess O/N-based functional groups. Their FTIR spectra also confirm the presence of C–O/C–N and C=O bonds (Fig. S6, ESI†).^31^,^33^

In previous reports, near zero or only a few percent (about 2%) N content has been found in CNDs obtained by the pyrolysis of templates confined in zeolites.^21^–^23^ Different from previous studies, the CNDs prepared in this work have highly intrinsic (about 22% based on XPS) N-containing groups. This further indicates that the carbonization process of the organic templates can be controlled by the template type, zeolite framework and calcination conditions, thus resulting in CNDs with different chemical compositions and surface groups, without adding any other reagents. Moreover, the highly N-doped surface may improve the hydrophilicity and stability of CNDs in aqueous systems, without the requirement for post-synthesis surface functionalization.

### Concentration-modulated luminescence

**Fig. 3:**
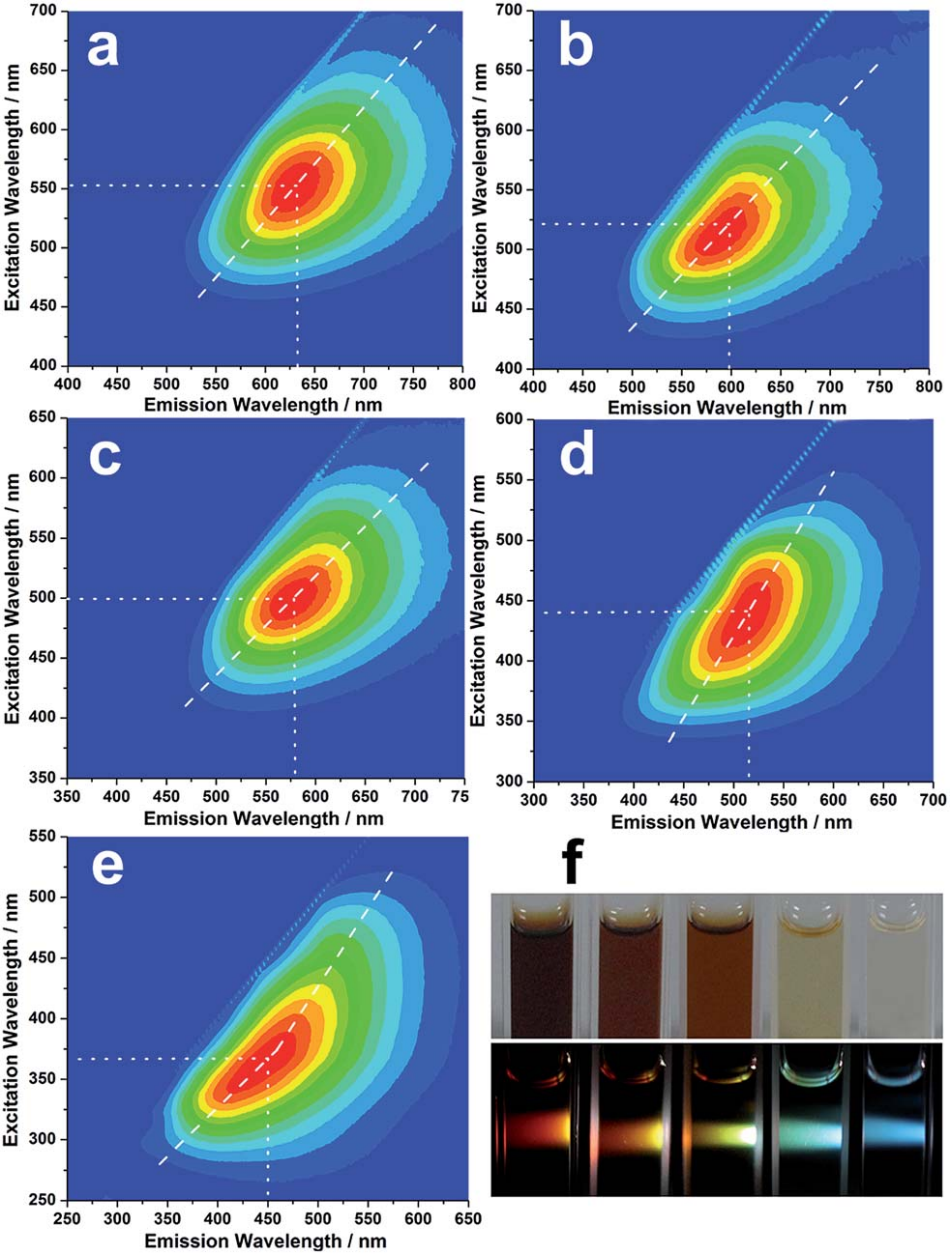
Excitation–emission matrix for (a) R-CNDs, (b) O-CNDs, (c) Y-CNDs, (d) G-CNDs and (e) B-CNDs in aqueous dispersions. Emission intensity rises from blue to green and to red; (f) optical photographs obtained upon irradiation of R-, O-, Y-, G- and B-CNDs with ambient (top image) and white (lower image) light.

Strikingly, the isolated CNDs not only exhibit excitation-dependent luminescence, but also display concentration-modulated luminescence. Fig. 3a–e show the excitation–emission matrices of the R-, O-, Y-, G- and B-CNDs. For each specific concentration, the emission wavelength red-shifts with increasing excitation wavelength, indicating the excitation-dependent fluorescence behavior of the CNDs. With a decrease in the concentration of the CNDs, the strongest emissions of these samples shift from 630 nm to 600 nm, 580 nm, 520 nm, and finally 450 nm, when their optimal excitation wavelength is varied gradually from 550 nm to 370 nm. As a result, five different colors, including red, orange, yellow, green and blue, are observed upon irradiation of these CND dispersions by white light, covering the entire visible spectrum (Fig. 3f). The highest fluorescence quantum yield of the CND solutions can reach *ca.* 18.7% (B-CNDs) without any further functionalization, which is higher than most of the bare and raw CNDs, which have typical values below 10%.

The as-made CND dispersions are stable for over six months, and no obvious PL changes are observed (Fig. S7, ESI†). Investigations into the cellular imaging and cellular toxicity indicate that the as-prepared CNDs are of low toxicity and biocompatible (Fig. S8, ESI†), suggesting their potential applications in biomedicine.

Notably, such concentration-modulated luminescence of CNDs across the entire visible spectrum is distinguished from previous reports, which tune emissions by controlling the reaction conditions or subsequent oxidation/reduction operations.^11^,^33^–^35^ Further experiments reveal that such concentration-modulated luminescence phenomena also occur for CNDs prepared by different methods (Fig. S9, ESI†). However, due to the discrepant structures and compositions of CNDs prepared by different methods, the stabilities of the CND dispersions are quite different, which may influence their luminescence behavior. A series of experiments were designed to gain a better understanding of the different fluorescence behaviors of CNDs at varying concentrations. In general, a fluorescent solution with a high concentration will lead to an inner filter effect. However, the asymmetric excitation–emission matrix of the B-CNDs (Fig. 3e) implies the existence of a self-absorption phenomenon. Inversely, with an increase in the CND concentration, such self-absorption weakens, as indicated by the relatively symmetric excitation–emission matrix of the R-CNDs (Fig. 3a). Thus, self-absorption might not be predominantly responsible for the concentration-modulated emission of CNDs.

The PL decay curves of the obtained CNDs were measured, and the decay fitting results are listed in Table S1.† Fig. S10† shows the PL decay curves of the B-CNDs probed at different emission wavelengths with excitation at 340 nm. The average lifetimes, which are obviously emission wavelength dependent, vary in the range of 4.10 to 6.52 ns. This indicates that multiple energy substates exist in B-CNDs.^38^ At high CND concentrations, the distance between two or multiple emitters is obviously decreased, which results in aggregation due to molecule interactions. This may result in the formation of a cluster that favors energy transfer between the emitters, which thus exhibit significant red-shifts.^38^ This is further confirmed by the TEM results, which show that the size of the B-CNDs (∼2.5 nm) is smaller than that of the R-CNDs (∼5.8 nm). Such clusters can be dissociated into isolated CND particles by solvent molecules at low concentrations. The detailed mechanism needs to be further investigated.

### pH-Modulated luminescence

**Fig. 4:**
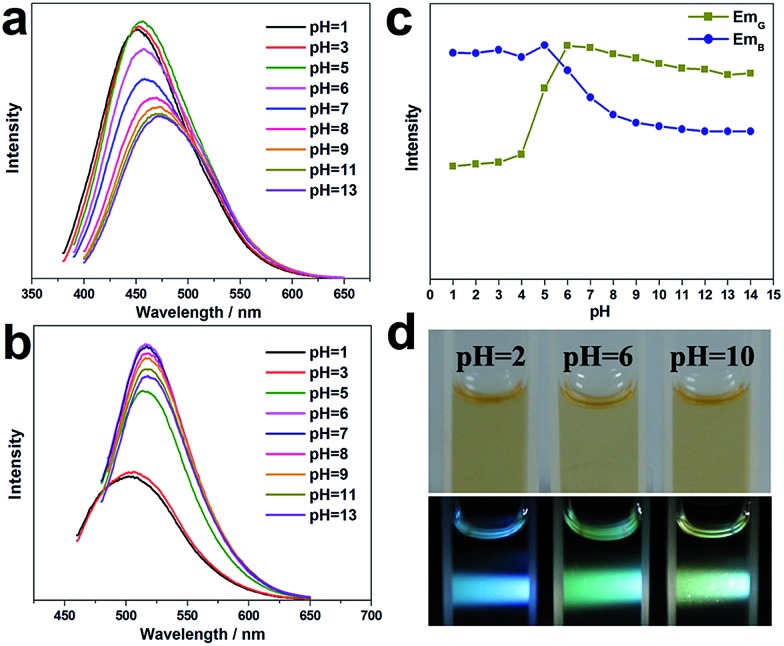
PL emission spectra of G-CNDs at various pH values excited by (a) 380 nm and (b) 460 nm UV light; (c) pH dependence of the maximum PL intensity of (a) and (b); (d) optical photographs obtained upon irradiation of CNDs at three different pH values with ambient (top image) and white (lower image) light.

The influence of the pH value on the PL properties of the CNDs has also been investigated. As shown in Fig. 4a, when excited by UV light at around 380 nm, the CND dispersions emit blue light at about 460 nm, and the PL intensity is unchanged from pH 1 to 5, but it decreases significantly at higher pH values (pH = 6–14). Strikingly, when excited by a wavelength of around 460 nm, the CND dispersions emit green-yellow light at about 525 nm. The intensity remains almost unchanged at high pH values of 6–14 but decreases obviously at lower pH values of 1–5 (Fig. 4b). Fig. 4c shows the change in absolute intensity of two PL emissions with varying pH value. Three different colors, *i.e.*, blue, green and green-yellow, can be observed at pH values of 2, 6 and 10 respectively, upon irradiation with white light (Fig. 4d). Previously, the modulation of the PL emission intensity of CNDs by pH value has been studied. However, pH-sensitive color tunable CNDs, which may offer a visualized and robust sensor for pH determination, have been rarely reported.

FTIR and UV-vis spectra of the CND dispersions at pH values of 2, 6, and 10 have been obtained (Fig. S11 and S12, ESI†). They show that the functional groups on the CNDs do not vary with pH value. We surmise that the carboxy and amino groups on the CND surfaces are sensitive to pH value and may change subtly in different acid or alkaline conditions, thereby affecting the optical properties of the CNDs.^33^,^39^ Understanding the origin of complex fluorescence definitely relies strongly on detailed physicochemical characterizations and the discovery of exact molecular structures.

## Conclusions

In summary, we have successfully developed fluorescent CNDs responsive to the concentration and pH value of their CND dispersions. The CNDs are prepared by the pyrolysis of *N*-methylpiperazine occluded in the MgAPO-44 zeolite. Abundant N/O-containing hydrophilic groups are *in situ* generated on the surface of the as-made CNDs, endowing these CNDs with excellent aqueous dispersibility and stability, as well as a high PL quantum yield of up to 18.7% without any further functionalization. These CNDs exhibit interesting concentration-dependent PL properties, including full-color emissions when excited by white light. Such tunable PL of CNDs might mainly result from the energy transfer between multiple emitters in the nanoclusters that form in the highly concentrated CND dispersions. Meanwhile, pH-sensitive color and PL intensity modulation of the CND dispersions have also been observed. The detailed mechanism needs to be further investigated. This work provides a new approach for facilely controlling the optical properties of CNDs through the pyrolysis of organo-templated zeolites, which may open more opportunities in the application of CNDs in multimodal sensing and full-color displays.

## Supplementary Material

Supplementary information
