# Supplementary material for: Carbogenic nanodots derived from organo-templated zeolites with modulated full-color luminescence
Source: Chem Sci. 2016 Feb 12;7(6):3564–8. doi: 10.1039/c6sc00085a (PMC6007353; doi:10.1039/c6sc00085a)
Supplement: Supplementary file 1 [file SC-007-C6SC00085A-s001.pdf]

Electronic Supplementary Material (ESI) for Chemical Science.
This journal is © The Royal Society of Chemistry 2016

Electronic Supplementary Information for:

Carbogenic Nanodots Derived from Organo-Templated Zeolites with Modulated Full-Color Luminescence

Ying Mu,[a] Ning Wang,[a] Zaicheng Sun,[b] Jing Wang,[a] Jiyang Li*[a] and Jihong Yu*[a]

[a]State Key Laboratory of Inorganic Synthesis and Preparative Chemistry, College of Chemistry, Jilin University, Changchun 130012, P. R. China. Fax: (+86) 431-85168608; E-mail: lijiyang@jlu.edu.cn; jihong@jlu.edu.cn

[b]Beijing Key Laboratory for Green Catalysis and Separation, Department of Chemistry and Chemical Engineering, Beijing University of Technology, Beijing, 100124, P. R. China

Preparation of Ref1-CNDs and Ref2-CNDs

Ref1-CNDs and Ref2-CNDs were prepared according to the reported references.[1,2] Ref1-CNDs: The fresh watermelon peel was firstly carbonized at 220 °C for 4 h under air atmosphere. The obtained product was dispersed in ultrapure water and sonicated for 30 min, and then centrifuged (10,000 ×g, 20 min). The supernate was filtrated with 0.2 μm filter membrane. Ref2-CNDs: Soy milk can be made at home with a soy milk machine, which was heated under autogenous pressure at 180 ºC in a 15 mL Teflon-lined stainless steel autoclave for 3h. The brown CNDs dispersion was centrifuged (10,000 ×g, 20 min) and filtrated with 0.2 μm filter membrane. The initially purified Ref1- and Ref2-CNDs were dull-red and had a concentration around 1.5 g L^{-1}. Then the solutions were quantificationally diluted to a concentration of 1.2 and 0.03 g L^{-1}.

1 C. Z. Zhu, J. F. Zhai, S. J. Dong, *Chem. Commun.* **2012**, *48*, 9367.

2 J. J. Zhou, Z. H. Sheng, H. Y. Han, M. Q. Zou, C. X. Li, *Materials Letters* **2012**, *66*, 222.

Cellular Toxicity and Cellular Imaging

Cellular toxicity test: Human epidermoid cancer cells (Hep2) were cultured first for 24 h in an incubator (37 ºC, 5% CO_2), and then kept for another 24 h by replacing the culture medium with 200μL of Iscove's Modified Dubecco's Medium (IMDM) that contains CNDs with different concentrations (0, 15, 30, 75, 100, 150, 200 μg/mL), respectively. Then, 20 μL of 5 mg/mL MTT (3-(4,5-dimethyl-2-thiazoly1) -2,5-diphenyl-2-H-tetrazolium bromide) solution was added to each cell well. The cells were further incubated for 4 h, followed by removing the culture medium with MTT. The resulting mixture was shaken for ca. 5 min at room temperature. The optical density (OD) of the mixture was measured at 490 nm. The cell viability was estimated according to the following equation: Cell Viability [%] = $(OD_{treated} / OD_{control}) \times 100$ % (Where $OD_{control}$ was obtained in the absence of CNDs, and $OD_{treated}$ obtained in the presence of CNDs.)

Cellular imaging: The cells were cultured in IMDM supplemented with 10% fetal bovine serum. Suspensions (200 μg/mL) of CNDs from the stock solution were prepared with Dulbecco's phosphate buffer saline (DPBS). After sonication for 10 min to ensure complete dispersion, an aliquot (typically 0.1 mL) of the suspension was added to the well of a chamber slide, then incubated at 37 ºC in a 5% CO2 incubator for 20 h. Prior to fixation of the cells on the slide for inspection with a confocal fluorescence microscope, the excess CNDs were removed by washing three times with warm DPBS.

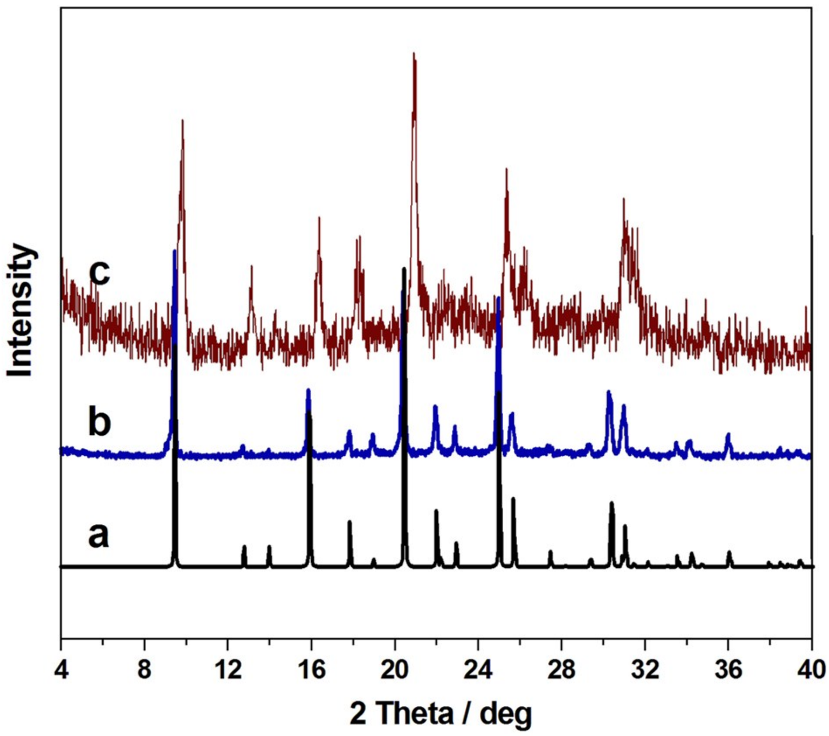

Fig. S1. Powder X-ray diffraction patterns of (a) simulated **CHA** zeolite and experimental (b) MgAPO-44 and (c) CNDs@ MgAPO-44.

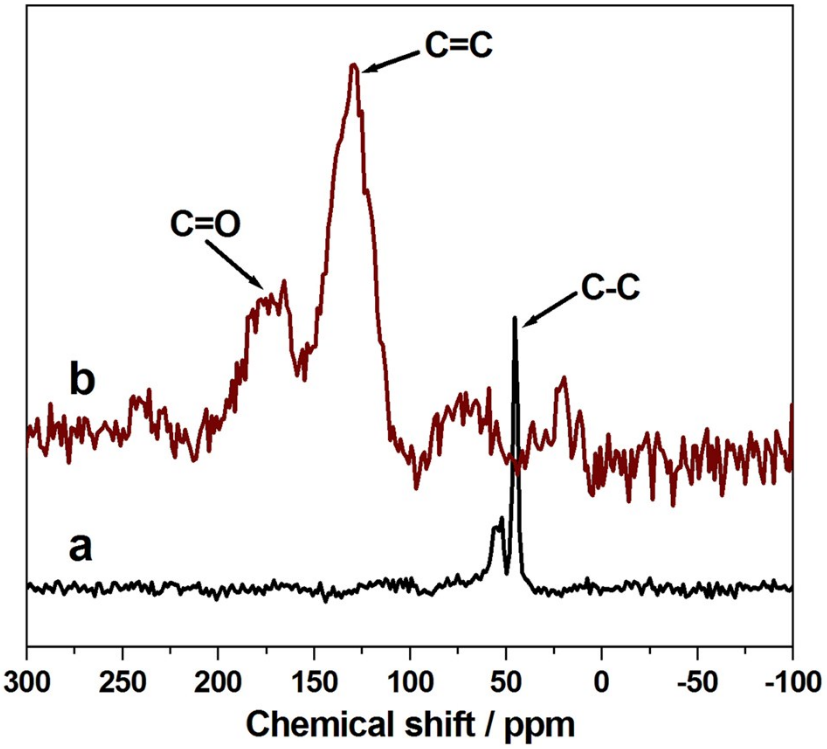

Fig. S2. ^{13}C MAS NMR spectra of (a) MgAPO-44 and (b) CNDs@MgAPO-44.

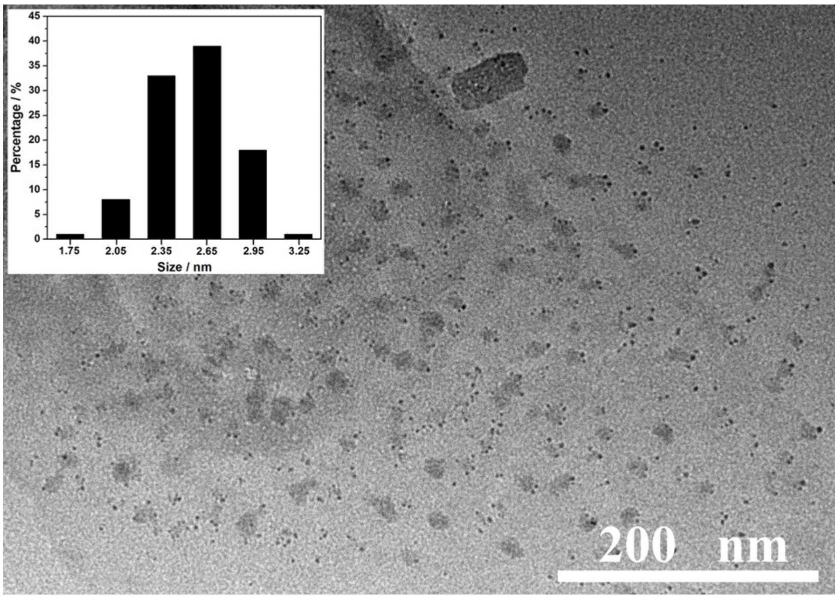

Fig. S3. TEM image of CNDs @ MgAPO-44 composite material. Inset image is size distributions of CNDs obtained by counting about 100 particles. The large particles observed are the fragments of MgAPO-44 framework, which may be formed during the treatments of calcination, ground and ultrasonication. These particles can be dissolved/removed during the alkaline treatment.

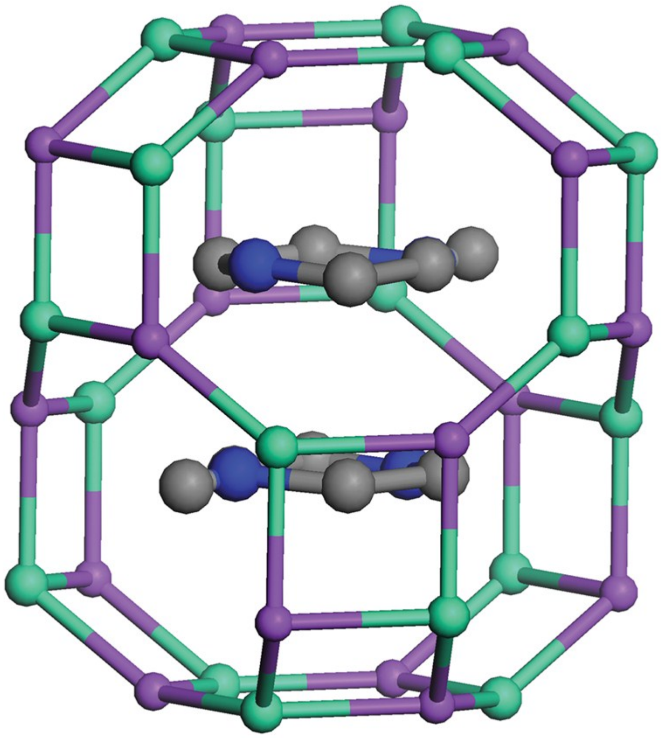

Fig. S4. CHA cage and schematic diagram of located *N*-methylpiperazine.

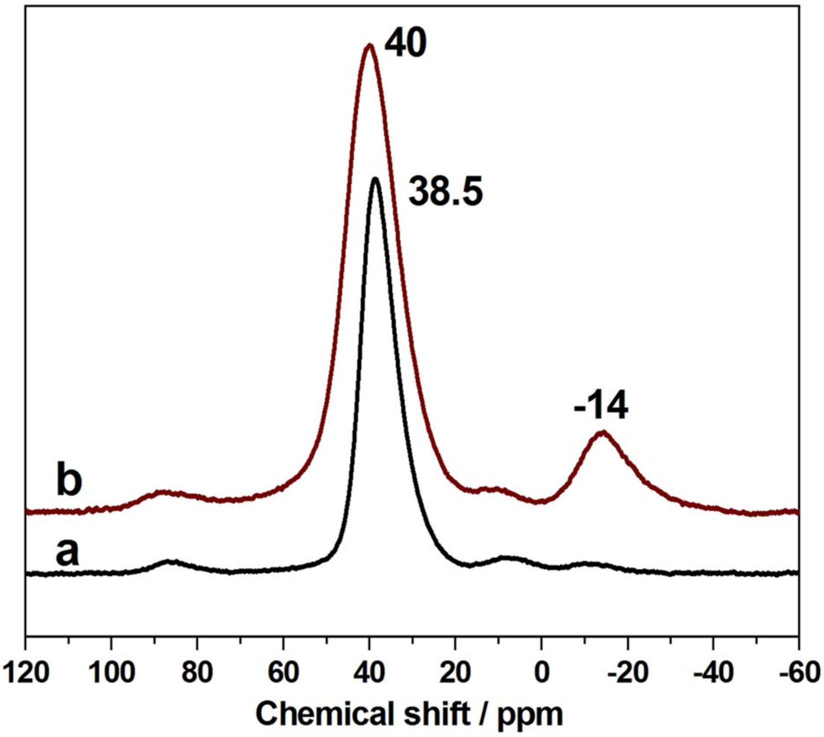

Fig. S5. ^{27}Al MAS NMR spectra of (a) MgAPO-44 and (b) CNDs@MgAPO-44.

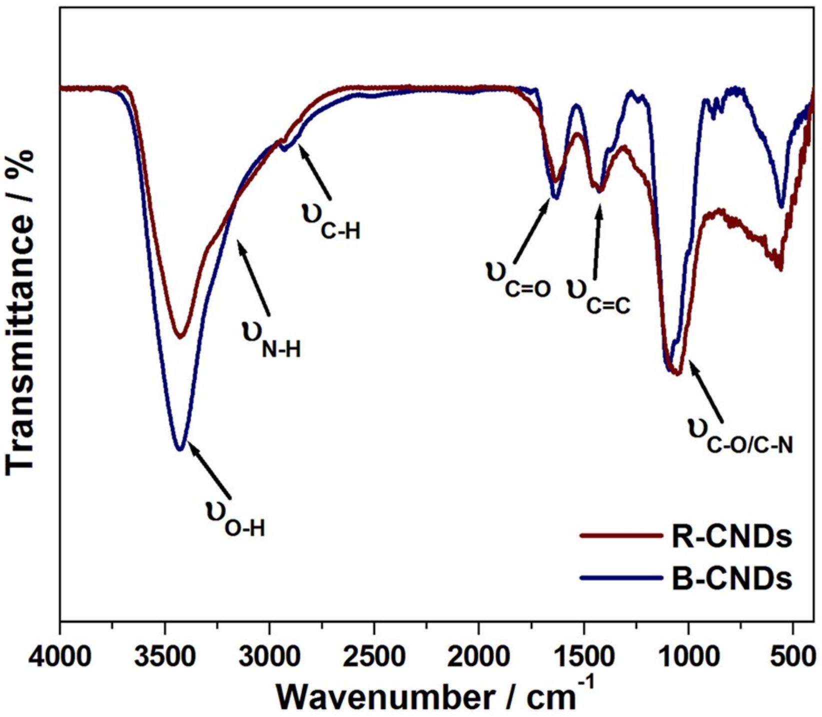

Fig. S6. FTIR spectra of R-CNDs and B-CNDs.

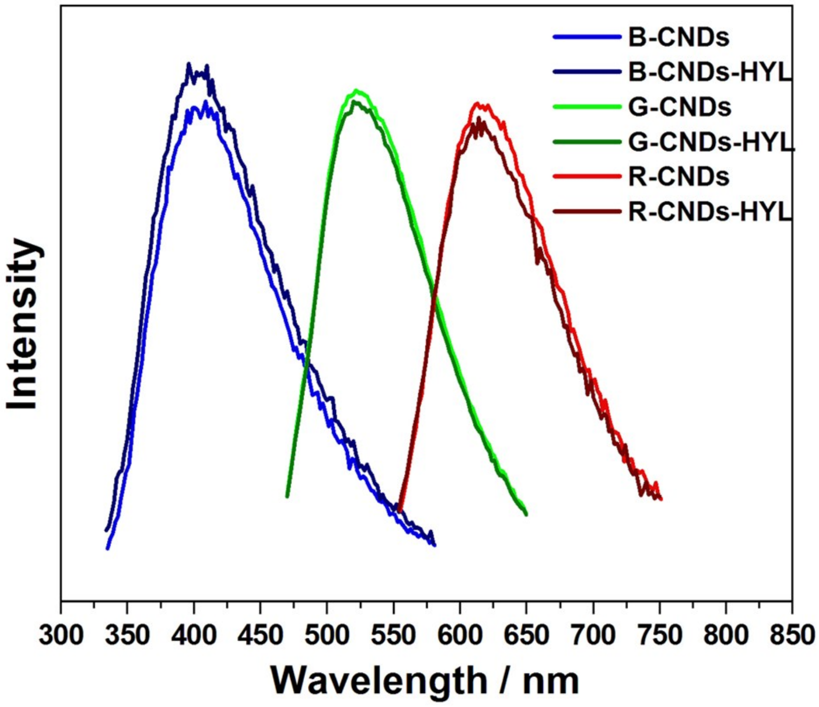

Fig. S7. PL emission spectra of as-prepared B-, G- and R-CNDs and these samples after six months (noted as B-, G- and R-CNDs-HYL, respectively).

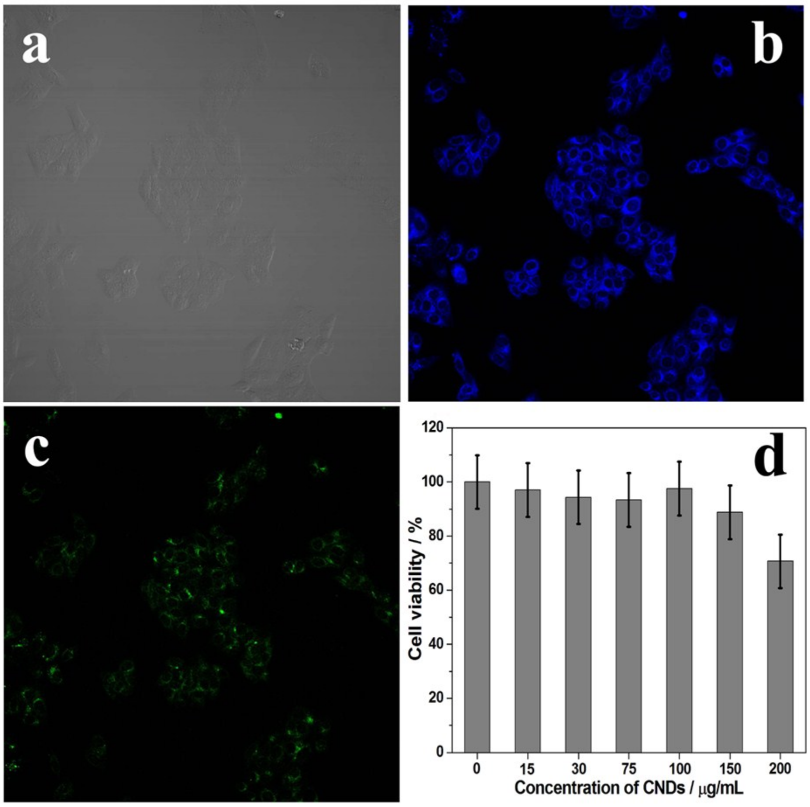

Fig. S8. Cellular imaging and cellular toxicity of CNDs. a), b) and c) washed cells images under bright field, 405 nm and 488 nm excitations, respectively; (d) effect of CNDs on Hep2 cells viability.

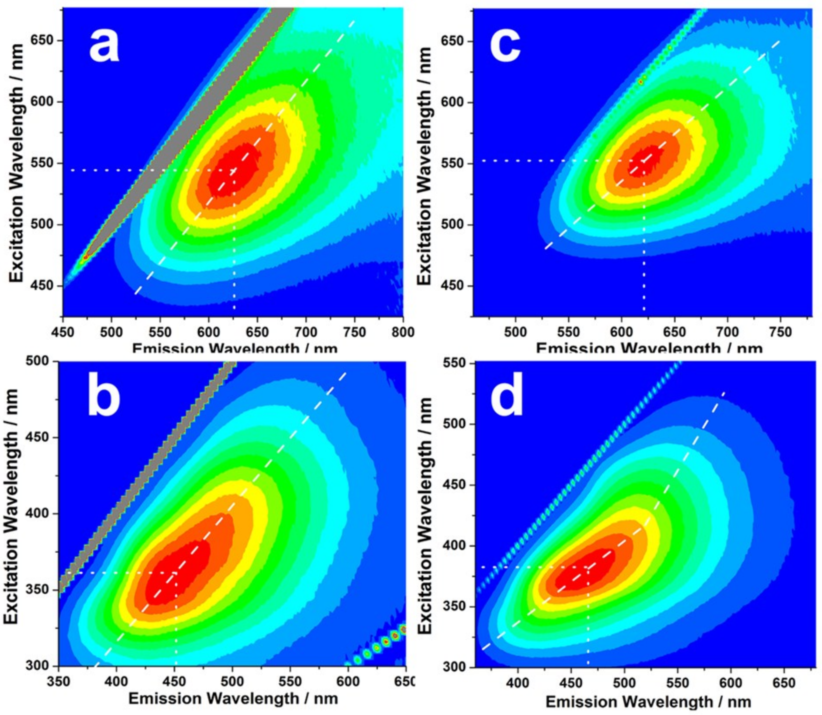

Fig. S9. Excitation-emission matrix for (a) Ref1-CNDs (1.2 g L^{-1}), (b) Ref1-CNDs (0.03 g L^{-1}), (c) Ref2-CNDs (1.2 g L^{-1}) and (d) Ref2-CNDs (0.03 g L^{-1}) in aqueous dispersions. Emission intensity raises from blue to green and to red. The luminescence of these two types of CNDs can also be tunable from red to blue by modulating their concentrations.

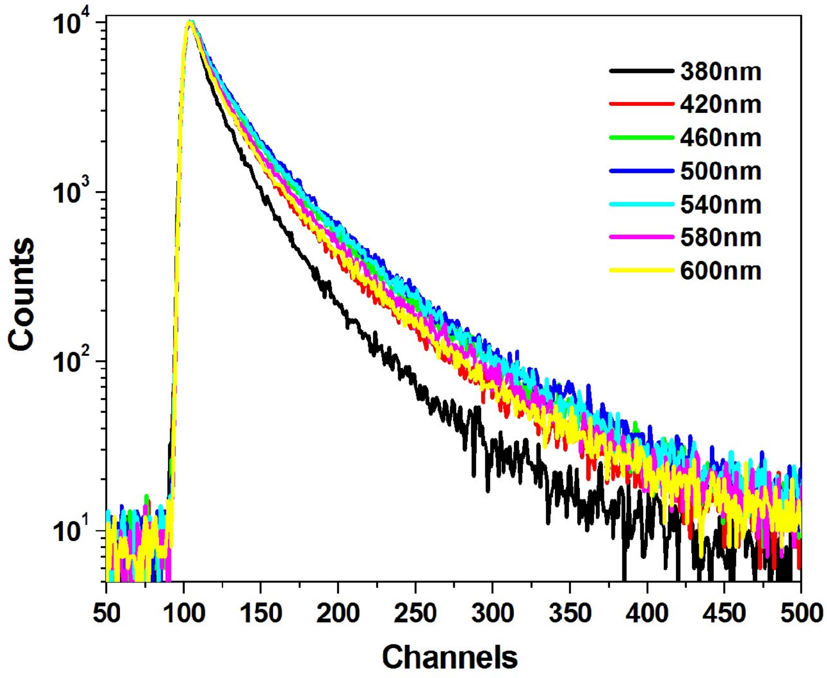

Fig. S10. PL decay curves of B-CNDs aqueous dispersions at 340 nm excitation detected at different emission wavelengths as indicated.

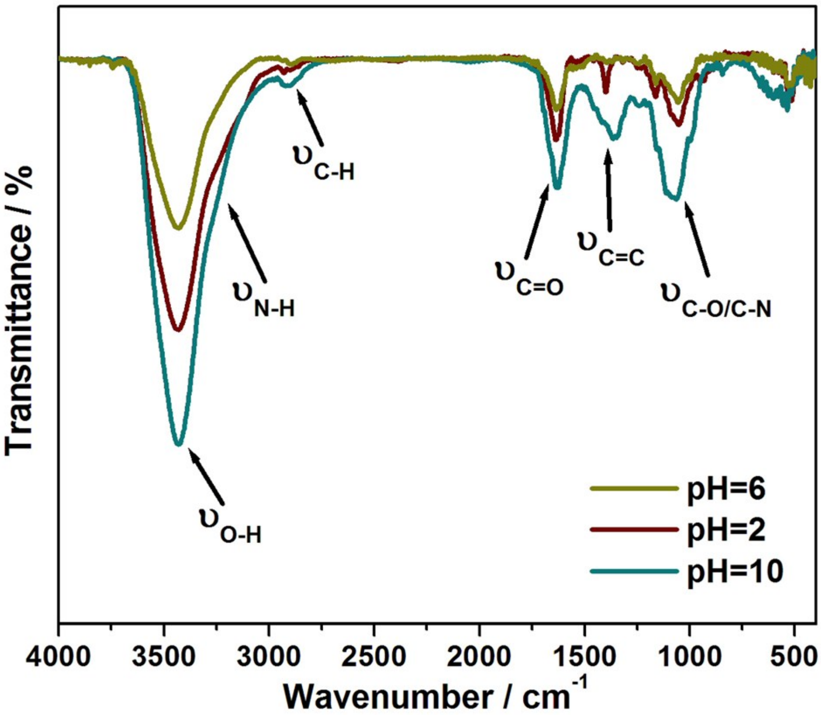

Fig. S11. FTIR spectra of G-CNDs at three different pH values.

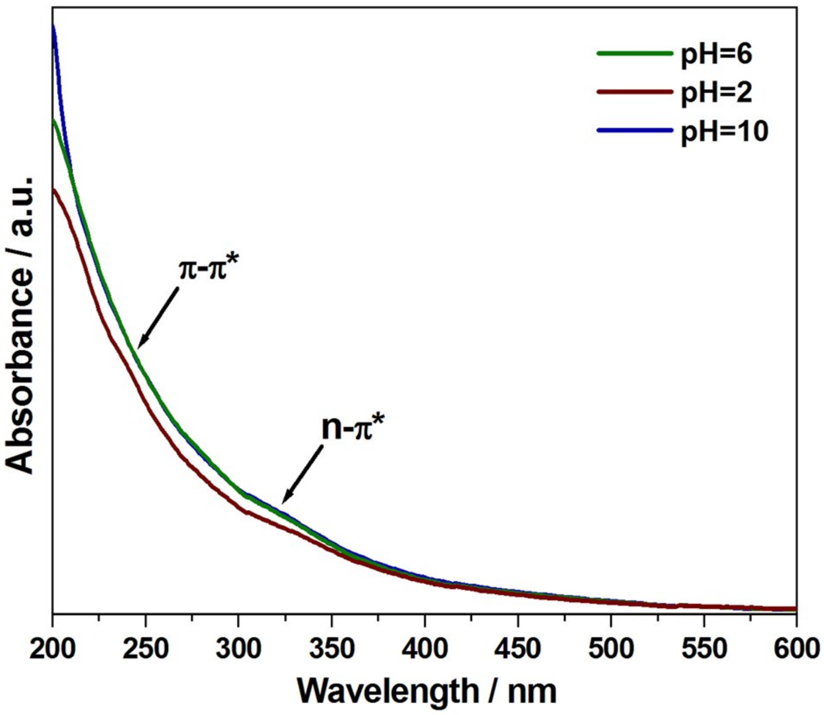

Fig. S12. UV-vis absorption spectra of G-CNDs at three different pH values.

Table S1. Fitting Parameters of the Corresponding PL Decay Curves

λ_{em} / nm	τ_1 / ns	B_1 / %	τ_2 / ns	B_2 / %	τ_3 / ns	B_3 / %	τ_{avg} / ns	CHISQ
380	1.89	49	5.35	46	14.27	5	4.10	1.21
420	2.10	40	5.74	49	14.87	11	5.25	1.07
460	2.65	43	6.89	45	16.03	11	6.10	1.02
500	2.87	42	7.25	45	15.88	13	6.52	1.07
540	2.27	32	6.02	50	14.40	17	6.26	1.10
580	2.17	40	6.30	47	14.99	12	5.68	1.10
600	2.04	42	6.09	47	15.04	11	5.35	1.26

λ_{ex} = 340 nm for B-CNDs
